# Exposure differences in railway noise, vibration, area-level socioeconomic conditions and migration density: spatial autocorrelation and urbanicity impacts in Southwest Sweden

**DOI:** 10.1007/s11356-026-37951-1

**Published:** 2026-07-02

**Authors:** Natalia Vincens, Jesper Löve, Kerstin Persson Waye, Mikael Ögren

**Affiliations:** 1 Sound Environment and Health, School of Public Health and Community Medicine, Institute of Medicine, University of Gothenburg, Gothenburg, Sweden; 2 School of Public Health and Community Medicine, Institute of Medicine, University of Gothenburg, Gothenburg, Sweden

**Keywords:** Railway noise, Railway vibration, Unequal exposure, Socioeconomic conditions, Migration density, Spatial autocorrelation, Urbanicity

## Abstract

Environmental exposures, such as railway noise and vibration, might be unequally distributed across communities. This study investigates how area-level socioeconomic conditions and migration density vary with the distribution of railway noise and vibration in Southwest Sweden. We employed linear regression and spatial autoregressive models to analyse data from the EpiVib study, which included 7,280 individuals living within 1 km of a trafficked railway in four regions of Southwest Sweden. The study assessed the relationships between exposure levels and area-level socioeconomic conditions and migration density using Statistics Sweden’s small-area division system (i.e., DeSO). Areas with lower socioeconomic conditions and higher migration density are exposed to higher levels of railway noise and vibration. Spatial autocorrelation analysis revealed that noise levels were higher in areas with more than 20% of inhabitants having a foreign background. The impact of area-level indicators on noise exposure varied with urbanicity, with higher exposure levels in rural and suburban areas compared to urban areas. Vibration exposure was more pronounced in areas with fair socioeconomic conditions and higher migration density. The study shows inequalities in railway noise and vibration exposure, according to area-level socioeconomic conditions and migration density. Areas with better socioeconomic conditions are consistently less exposed to both railway noise and vibration. Furthermore, the impact of area-level indicators on noise exposure depends on urbanicity levels. Future research, interventions and policies that account for area-level socioeconomic factors as well as urbanicity are advised to ensure environmental equity and justice regarding rail traffic exposures.

**Supplementary Information:**

The online version contains supplementary material available at 10.1007/s11356-026-37951-1.

## Background

Addressing environmental inequities is critical for promoting a healthy and just society. Disadvantaged communities often face worse environmental conditions, hindering sustainable development and increasing health disparities (European Environment Agency 2018). In the context of sustainability, rail traffic has increased as a better alternative transportation mode for climate reasons. Yet, although more research is needed, studies suggest that railway noise and vibration might negatively affect health, including annoyance, sleep disturbances, atrial fibrillation, stroke and diabetes (Fu et al. 2023; Seidler et al. 2023, 2018; Vienneau et al. 2024; WHO 2018). Estimates suggest that in Europe, 22 million people are exposed to high levels of railway noise (> 55 dB Lden) (European Environment Agency 2020), although these figures are conservative. In addition, health impact assessments often exclude synergistic exposures associated with rail traffic (e.g., vibration) and potential social inequities in exposure.

Compared to other environmental exposures, such as air pollution, transportation noise has been less studied with regard to inequalities. Indeed, findings are inconsistent: while some studies report that more deprived groups face higher levels of noise, others find greater exposure among individuals in higher socioeconomic positions and in affluent areas (Dreger et al. 2019; Trudeau et al. 2023). These conflicting findings likely reflect variations in the transportation noise source (e.g., road, rail, or air traffic), contextual factors (e.g., urban planning, transport policy, housing policies, and residential choices), as well as methodological approaches (e.g., choice of socioeconomic indicator, level of analysis, and statistical models) (Dreger et al. 2019; Trudeau et al. 2023). Studies using single indicators of material resources such as income and area-level socioeconomic factors more consistently report higher noise exposure with increased economic deprivation (Dreger et al. 2019; Trudeau et al. 2023). In addition, results differ whether inequalities are analysed at individual- or area-level, as these capture different dimensions of social vulnerability. Methodological choices also matter. For example, studies comparing ordinary least squares (OLS) linear regression with spatial regression models have reported different findings (Verbeek 2019).

Still, in general, research increasingly points to railway noise as particularly associated with socioeconomic disadvantages. A Dutch study found railway and aircraft noise to be more unequally distributed than road traffic noise (Kruize et al. 2007), and recent studies in England suggest that railway noise might be the source of transportation noise most strongly related to area-level socioeconomic deprivation (Peris and Arguelles 2023). Studies using individual- and neighbourhood-level indicators in other contexts have similarly found higher noise exposure in lower-income or more deprived communities, including in North America and large European cities (Casey et al. 2017; Tonne et al. 2018).

Importantly, railway noise and vibration follow a clear geospatial pattern primarily shaped by the location of the rail tracks, leading to clusters of observations where neighbouring dwellings and areas share similar exposure levels. In addition to physical propagation factors, area-level socioeconomic conditions and migration density may further contribute to these spatial patterns through residential sorting and urban planning processes. As a result, both environmental exposures and key social indicators exhibit spatial autocorrelation, violating the independence assumption of conventional linear regression models. Studies that ignore these spatial processes risk biased estimates and misleading conclusions regarding environmental inequities. Spatial regression models explicitly account for spatial dependence and have therefore been frequently used in environmental equity research (Carrier et al. 2016; Elford and Adams 2021; Verbeek 2019).

Railways have played a central role in Sweden’s development since the nineteenth century (Haikola and Anshelm 2022). Over the years and with expansions, rail traffic has increasingly generated growing concerns about noise and vibration (European Environment Agency 2025). Regulatory action emerged in the late 1990s, with the Environmental Code in 1998 (Sveriges riksdag 1998) and subsequently the EU directives in 2000s mandating noise mapping and mitigation plans (European Parliament 2002). Sweden has since established national guidelines that set reference values for noise and also vibration, integrating health considerations more comprehensively than many European counterparts (Naturvårdsverket 1999; Trafikverket 2017). In Europe, current policy aims to halve harmful traffic noise exposure by 2030 (European Environment Agency 2025), while systematic strategies for vibration control remain in comparison less developed (Naturvårdsverket 1999). Mitigation measures such as barriers, insulation, and source improvements demonstrate policy responsiveness in Sweden, yet questions persist about how equitably these protections are distributed across affected communities (European Environment Agency 2025).

The railway corridors included in this study traverse heterogeneous geographic and land-use contexts across Southwest Sweden, reflecting the long-standing role of rail infrastructure in regional development and settlement patterns (Haikola and Anshelm 2022). In urban and suburban areas, railways frequently run through built environments with mixed residential and commercial land use, often near transport hubs. In contrast, rural railway segments typically pass through lower-density residential areas and agricultural or forested landscapes, where detached housing is more common and alternative transport infrastructure is more limited. Across these contexts, residential dwellings are often located close to railway tracks, partly due to historical settlement processes and housing development shaped by transport infrastructure (Gibbons and Machin 2005). These differences in land use, building typology, and settlement structure influence both the propagation of railway noise and vibration and the spatial distribution of populations exposed to these environmental stressors (European Environment Agency 2018; Trafikverket 2017).

In this context, environmental equity is defined in this project according to the social vulnerability model by Diderichsen et al. (2019) as the extent to which environmental burdens are fairly distributed across population groups, and whether differences in exposure are compounded by unequal susceptibility to their effects and unequal capacity to cope with or mitigate those exposures. In the context of railway noise and vibration, environmental inequity may therefore reflect not only disproportionate exposure among certain socioeconomic or migration-related groups, but also differences in health susceptibility, housing conditions, and access to mitigation or decision-making processes (Vincens et al. 2026). In the present analysis, we however focus on distributional aspects of environmental equity to examine how area-level socioeconomic conditions and migration density influence the distribution of railway noise and vibration, exploring different statistical approaches. We also explored urbanicity as a potential effect modifier in these relationships.

## Method

### Data source and study population

Our analyses used data from the EpiVib study (Maclachlan et al. 2018), which surveyed a randomly selected sample of residents living close to a railway in Halland, Västra Götaland, Värmland, and Örebro regions in Southwest Sweden in 2017. The selected study areas were populated regions within 1 km of active railways (i.e., at least ten passing freight trains daily and nightly), with vibration measurements available from several dwellings. Areas with nearby major motorways or significant air traffic were excluded from the sampling frame to ensure that railway noise was the primary transportation noise source.

Between March and June 2017, up to two residents per household, aged 18–80, residing in the specified areas, were invited to participate in the study. A total of 35,011 individuals received postal questionnaires, with two reminder mailings issued. The response rate was 20.8%, with 7280 participants completing and returning the questionnaire. The study followed the principles of the Helsinki Declaration, received approval from an ethical review board, and required signed informed consent from all participants.

A comprehensive postal/online questionnaire was used as part of the EpiVib project to recruit participants in residential locations in proximity to the railway network (Maclachlan et al. 2018). The questionnaire collected information on several dimensions including sociodemographic characteristics, health, and perceptions related to railway noise and vibration. However, individual-level questionnaire responses were not used in the present analysis. Instead, the questionnaire served as a sampling and geocoding tool, enabling the linkage of participants’ residential addresses to modelled railway noise and vibration exposure and to area-level socioeconomic indicators using Geographic Information Systems (GIS). Area-level socioeconomic conditions and levels of migration density were assessed based on Statistics Sweden’s small-area division system known as DeSO, which divides Sweden into 5,984 regions with populations ranging from 700 to 2700 residents (SCB–Statistikmyndigheten n.d.).

### Variables

#### Outcomes: railway noise and vibration

##### Noise

Railway noise exposure was calculated as the equivalent continuous A-weighted sound pressure level (LAeq) at the most exposed façade, using the Nordic prediction method (Naturvårdsverket 1999). We used the day-evening-night level (Lden), which was derived from LAeq levels during the day, evening, and night, with added penalties of 5 dB for the evening period (19:00–22:00) and 10 dB for the night period (22:00–07:00). Railway noise was analysed both as a continuous variable and as a dichotomous variable, categorised into potentially harmful and non-harmful exposure levels. A threshold of 55 dB Lden was applied to define harmful exposure, in accordance with the WHO Environmental Noise Guidelines for the European Region (WHO 2018).

##### Vibration

Railway vibration exposure was estimated through an empirical calculation model based on site-specific vibration measurements combined with geological data. Detailed methods for these calculations are discussed in previous work (Ögren et al. 2021). Vibration exposure was quantified as the peak weighted vibration velocity at the building foundation (Vmax), reported in mm/s. For analysis, railway vibration was treated both as a continuous and as a dichotomous variable, distinguishing between potentially harmful and non-harmful exposure levels. A cut-off value of 0.3 mm/s was applied, based on findings from an experimental study indicating that acute physiological responses to vibration can occur at this level, potentially leading to long-term health effects (Smith et al. 2015).

#### Socioeconomic indicators

##### Area-level socioeconomic conditions

Area-level socioeconomic conditions were calculated using a socioeconomic index for the included DeSO areas (*n* = 119 in our sample; median of 47 respondents per DeSO area). This index was calculated based on the average of the proportion of individuals with (i) low economic standard (i.e., disposable income lower than 60 percent of the median income), (ii) low education level (i.e., compulsory education – 9 years education or less), and (iii) long-term unemployment (> 6 months) or receiving financial welfare support for at least 10 months (Delegationen Mot Segregation (DELMOS) 2021; SCB–Statistikmyndigheten n.d.). This index was then used to classify areas into 4 groups as follows: areas facing socioeconomic challenges (≥ mean SES index + 1 SD), areas with fair socioeconomic conditions (mean SES index + 0 SD and < mean SES index + 1 SD); areas with good socioeconomic conditions (≥ mean SES index - 1 SD and < mean SES index + 0 SD); areas with very good socioeconomic conditions (< mean SES index - 1 SD).

##### Area-level migration density

We used the proportion of individuals with foreign background (i.e., born outside Sweden and in Sweden with two foreign-born parents) at DeSO level (SCB – Statistikmyndigheten n.d.) as an indicator for migration-related residential segregation. The proportion of individuals with foreign background varied from 3.6 to 51.1% with a mean of 15.4 (SD 9.5). The indicator “migration density” was operationalised as a categorical variable: less than 10%, 10–20% and higher than 20% with foreign background.

#### Urbanicity

DeSO areas were classified as urban, suburban and rural according to criteria from Statistics Sweden. Rural areas are defined as the areas located mostly outside major population concentrations or agglomerations. Suburban areas are mostly located in a population concentration or urban area, but not in the municipality’s central city and urban areas are mostly located in the municipality’s central city. In Sweden, 17% of DeSO areas are rural, 10% are suburban and 73% are urban (SCB – Statistikmyndigheten n.d.).

### Analysis

We estimated the associations between area-level socioeconomic conditions and migration density with the participants’ exposure levels to railway noise and vibration velocity using different regression models. We selected reference categories for the categorical indicators based on expectations of lower exposure levels among privileged areas (i.e., very good socioeconomic conditions and lower migration density). In Tables 2 and 3, we used OLS linear regression for both noise and vibration in models 1 A and 1B for socioeconomic conditions and migration density, respectively. These models have an independence assumption that overlooks that observations might be clustered and spatially distributed. We used a Moran’s I statistic test to assess spatial autocorrelation. Significant spatial autocorrelation supported the use of spatial regression models.

To account for spatial dependence, we estimated spatial autoregressive (SAR) models (Models 2 A and 2B) using maximum likelihood methods. Spatial relationships were defined using a row-standardized inverse-distance spatial weights matrix based on participants’ residential coordinates, such that geographically closer observations exert greater influence. Duplicate coordinate points were minimally adjusted to enable matrix construction. The inverse-distance specification was selected to reflect the distance-decay pattern of railway noise and vibration propagation. The SAR models included both a spatially lagged dependent variable and a spatially autocorrelated error term, allowing us to account for spatial dependence in exposure levels as well as in unobserved factors. We report total effects, combining direct and indirect (spillover) effects of area-level indicators on the outcomes. For interaction analyses, we examined effect modification by urbanicity (urban versus rural/suburban areas) using linear regression models. This approach was chosen due to computational complexity and convergence challenges associated with estimating interaction effects within spatial autoregressive frameworks and may not fully capture spatial dependence in interaction terms. Lastly, we used logistic regression models to investigate the influence of area-level indicators on exposure ≥ 55 dB Lden and ≥ 0.3 mm/s Vmax.

We report predicted exposure levels, beta-coefficients and 95% CI for the linear regression models. For the SAR models, we report the total effects regression coefficients combining the direct and indirect effects (i.e., spillover effects) of area-level indicators on the outcomes. For the logistic regression we report predicted probabilities, OR and 95% CI. Predicted probabilities derived from a logistic regression analysis were used to estimate the impact of area-level indicators on exposure levels of noise and vibration velocity considered potentially detrimental to health. All analyses were performed in Stata 18.

## Results

Table 1 provides descriptive statistics of the study sample. Participants are on average 56 years of age with both males and females represented. The vast majority live in urban and suburban areas although over 13% live in rural areas. Participants live on average 411.5 m from a trafficked railway and are overall exposed to noise levels ranging from 38.1 to 77.4 dB Lden and vibration velocity ranging from 0 to 3.5 mm/s Vmax. Noise and vibration are positively correlated (Spearman's 0.70; *p* = 0.000) although being exposed to Lden ≥ 55 dB and Vmax ≥ 0.3 mm/s is not as correlated (Spearman's 0.18; *p* = 0.000). Regarding the area-level indicators, area-level socioeconomic conditions and area-level migration density are negatively correlated (Spearman's −0.52; *p* = 0.000), with areas with better socioeconomic conditions correlated with areas with lower migration density. Participants were represented across all 100 m distance categories within the 0–1000 m buffer from the railway, with proportions ranging from 5.4% in the 900–1000 m category to 16.3% in the 200–300 m category (Supplementary Table S1). The distribution of respondents along the railway corridor was uneven, which is consistent with variation in settlement density across different urbanicity contexts, as reflected in the distribution of participants across urban, suburban, and rural areas (Table 1).

**Table 1:** Descriptive statistics of the distribution of study participants’ exposure levels to railway noise and vibration according to area-level socioeconomic conditions and migration density (*n* = 7280)

	*N* = 7280	Area-level socioeconomic conditions				Area-level migration density		
		Challenges *n* = 422	Fair *n* = 1497	Good *n* = 4829	Very good *n* = 532	< 10% foreign background *n* = 2352	10–20% foreign background *n* = 3439	> 20% foreign background *n* = 1489
	% or mean(SD)	% or mean(SD)	% or mean(SD)	% or mean(SD)	% or mean(SD)	% or mean(SD)	% or mean(SD)	% or mean(SD)
Age, years	56.0 (15.6)	59.3 (14.9)	57.9 (15.6)	55.3 (15.6)	54.8 (15.4)	55.7 (15.4)	56.2 (15.8)	56.3 (15.5)
Sex								
Female	50.7	51.4	50.7	48.8	47.9	52	50.1	50.1
Rural–urban divide								
Rural	13.8	0	22.8	13.8	0	29	9.4	0
Suburban	9.1	29.9	19.9	0.6	39.1	10.1	12.3	0
Urban	77.1	70.1	57.3	85.6	60.9	60.8	65.9	100
Distance to the railway (m)	411.5 (261.2)	409.1 (251.6)	354.6 (254.9)	421.6 (259.6)	482 (272.9)	425.2 (260.5)	398.5 (255.4)	419.8 (274)
Railway noise (Lden- dB)	58.6 (6)	59.5 (5.3)	59.1 (5.6)	58.6 (6)	56.7 (6.5)	58.8 (5.9)	58.5 (6)	58.5 (6.1)
Railway vibration speed (Vmax (P50 – P25-P75))	0.02 (0.00–0.05)	0.01 (0.00–0.04)	0.03 (0.00–0.11)	0.01 (0.00–0.05)	0.00 (0.00–0.01)	0.01 (0.00–0.04)	0.01 (0.00–0.06)	0.02 (0.00–0.09)

**Fig. 1:**
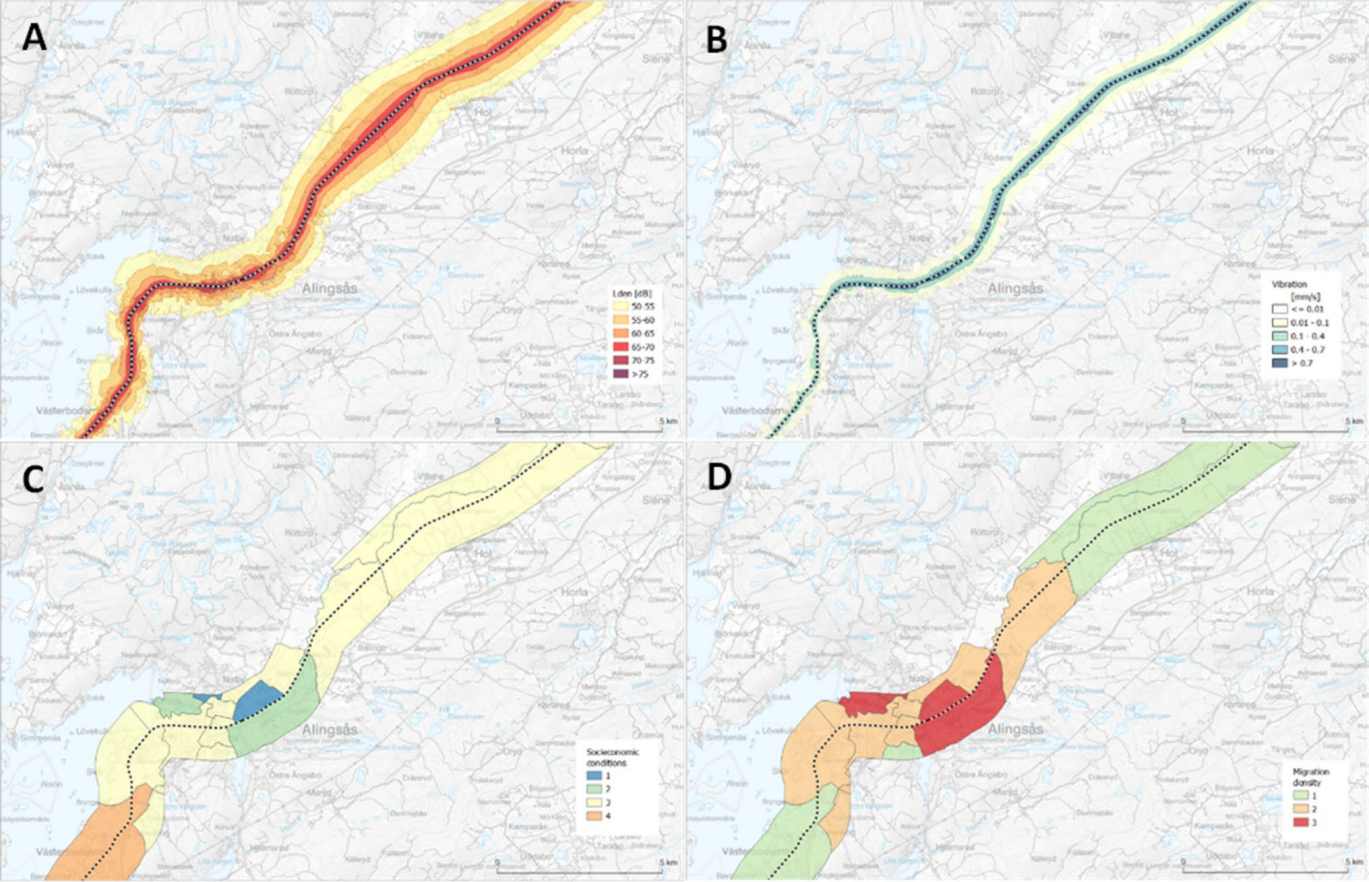
Maps of a railway segment illustrating the distribution of (**A**) noise, (**B**) vibration, (**C**) area-level socioeconomic conditions and (**D**) area-level migration density

Maps of a railway segment exemplify the distribution of noise and vibration exposure as well as area-level socioeconomic conditions and migration density (see Fig. 1). These maps illustrate the difference between noise and vibration propagation, highlighting the complexities in environmental exposures related to rail traffic for instance, with discontinuities in vibration velocity (likely due to soil characteristics) but not in noise levels even very close to the tracks. We can also see an overlap of high noise and vibration exposures in areas with fair and challenging socioeconomic conditions as well as higher migration density. Yet, areas outside towns (Allingsås in this segment) that are characterised at the area-level by good and very good socioeconomic conditions and lower migration density are not exempt from high exposure levels closer to the rail tracks.

**Fig. 2:**
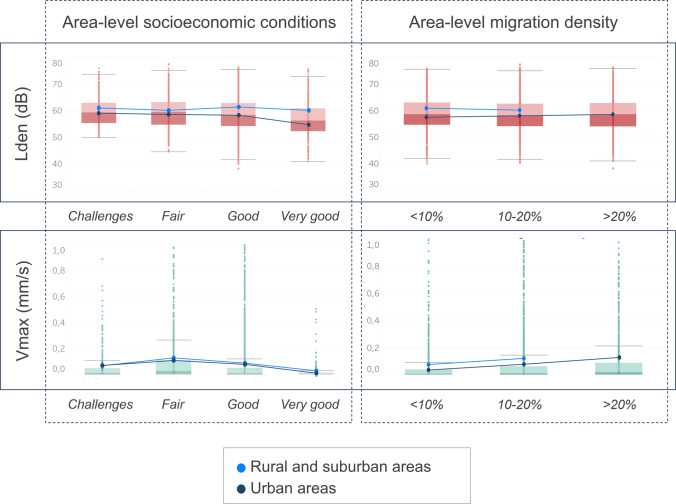
Boxplots for the distribution of noise and vibration according to (i) area-level socioeconomic conditions and (ii) area-level migration density. Connected lines represent the predicted exposure values following the interaction analysis on the impact of area-level indicators on noise and vibration according to urbanicity classes (rural-suburban versus urban areas)

Figure 2 illustrates the relationship between area-level socioeconomic conditions and migration density on the distribution of noise and vibration. Residents in areas with better socioeconomic conditions are, on average, exposed to lower levels of noise and vibration velocity. However, even in areas with good and very good socioeconomic conditions, a significant portion of participants is exposed to noise levels considered detrimental to health (Lden ≥ 55 dB). The boxplots in Fig. 2 are descriptive and illustrate the distribution of noise and vibration exposure across categories. Formal assessment of differences between categories is provided by the regression analyses presented in Tables 2 and 3, which indicate that the visual contrasts in Fig. 2 correspond to statistically significant differences for some category comparisons.

**Table 2:** Regression analysis for the associations between study participants’ residential exposure to railway noise/vibration and area-level socioeconomic conditions (*n* = 7280)

	Noise (Lden)		Vibration (Vmax)	
	Model 1A	Model 2A	Model 1A	Model 2A
	Linear regression	SAR	Linear regression	SAR
	Coef. (95% CI)	Coef.*(95% CI)	Coef.(95% CI)	Coef.*(95% CI)
Area-level socioeconomic conditions				
Socioeconomic challenges	2.81 (2.05; 3.58)	1.78 (0.94;2.62)	0.05 (0.02; 0.08)	0.06 (0.03; 0.09)
Fair socioeconomic conditions	2.42 (1.83; 3.01)	1.66 (1.01;2.30)	0.10 (0.08; 0.12)	0.10 (0.08; 0.13)
Good socioeconomic conditions	1.90 (1.36; 2.43)	1.24 (0.67;1.81)	0.06 (0.04; 0.08)	0.06 (0.04; 0.08)
Very good socioeconomic conditions	Ref	Ref	Ref	Ref
Moran’s I test	1822.74 *p* < 0.001		1839.23 * p* < 0.001	
Autocorrelation				
Area-level indicator		0.00		0.00
Outcome		0.00		0.00
Error		0.00		0.00
AIC	46,641.93	44,054.98	−1786.979	−4586.86
BIC	46,669.50	44,144.59	−1759.407	−4497.26

*SAR*  spatial autoregressive models; *Coef*  coefficient; *CI*  confidence interval; *AIC*  Akaike information criteria; *BIC* = Bayesian information criteria

*Coefficients for the total effect (direct plus indirect estimates)

**Table 3:** Regression analysis for the associations between study participants’ residential exposure to railway noise/vibration and migration density (*n* = 7280)

	Noise (Lden)		Vibration (Vmax)	
	Model 1B	Model 2B	Model 1B	Model 2B
	Linear regression	SAR	Linear regression	SAR
	Coef. (95% CI)	Coef.* (95% CI)	Coef. (95% CI)	Coef.* (95% CI)
Area-level migration density				
< 10% foreign background	Ref	Ref	Ref	Ref
10–20% foreign background	−0.33 (−0.65;−0.02)	−0.03 (−0.36;0.31)	0.03 (0.02;0.05)	0.04 (0.03;0.05)
> 20% foreign background	−0.28 (−0.67;0.11)	0.50 (0.06;0.93)	0.08 (0.06;0.09)	0.09 (0.07;0.10)
Moran’s I test	1824.71 *p* < 0.001		1825.49 *p* < 0.001	
Autocorrelation				
Area-level indicator		0.00		0.00
Outcome		0.00		0.00
Error		0.00		0.00
AIC	46,710.51	44,143.26	−1819.35	−4620.24
BIC	46,731.19	44,225.98	−1798.67	−4537.53

*SAR*   spatial autoregressive models; *Coef*   coefficient; *CI*   confidence interval; *AIC*   Akaike information criteria; *BIC*   Bayesian information criteria

*Coefficients for the total effect (direct plus indirect estimates)

Standard linear regression analysis suggests that in areas with better socioeconomic conditions, noise levels and vibration velocity are lower. In the case of noise, we observe a clear trend with decreasing exposure levels following improving socioeconomic conditions with average differences up to 2.8 dB Lden between areas with social challenges and areas with very good socioeconomic conditions. Although such a difference may not be perceived as a large change by individuals in all situations, it represents a substantial shift (i.e., almost double the sound pressure level) in long-term environmental noise exposure at the population level. For vibration, higher velocity is associated with areas with fair socioeconomic conditions and not areas facing challenges (see Table 2). Regarding linear regression models for area-level migration density, we observe opposite effects for noise and vibration: areas with a higher migration density are associated with higher vibration velocity but not with higher noise levels (see Table 3).

Moran’s I test after fully adjusted models suggests substantial spatial autocorrelation. Model 2 in Tables 2 and 3 addresses this issue using spatial lags for railway noise and vibration, predictors, covariates and residuals. Model 2 estimates are similar to the linear regression analysis for both noise and vibration, with regards to area-level socioeconomic conditions. In the analysis using area-level migration density we observe a similar trend as the linear regression for vibration. For noise, SAR models differ from linear regression models, with slightly higher exposure levels with higher migration density. Findings support significant spatial autocorrelation effects not only for the residuals but also for rail traffic exposure and area-level socioeconomic conditions and migration density. Model diagnostics suggest better fit (smaller AIC and BIC) for the SAR model regarding both outcomes and predictors.

**Fig. 3:**
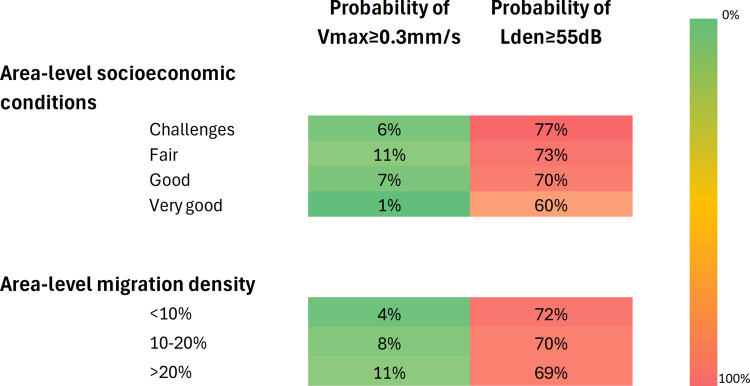
Colour-coded table of the predicted probabilities of exposure to noise ≥ 55 dB and vibration ≥ 0.3 mm/s Vmax. Probabilities were predicted based on logistic regression models

Figure 3 shows the predicted probability of being exposed to Lden ≥ 55 dB: 60% in very good areas and 77% in areas facing socioeconomic challenges. For vibration, although we observe, on average, low Vmax in the entire sample, we report disparities in exposure according to socioeconomic conditions. The predicted probability of being exposed to Vmax ≥ 0.3 mm/s is 1% in very good areas and 11% in areas with fair socioeconomic conditions.

Area-level migration density had almost no influence on the distribution of noise levels, including the probability of being exposed to Lden ≥ 55 dB (see Figs. 2 and 3). However, it did influence the vibration exposure distribution, with higher vibration velocity and a higher probability of exposure to Vmax ≥ 0.3 mm/s in areas with more than 20% of inhabitants with a foreign background.

An interaction analysis shows that associations between area-level indicators and noise depend on urbanicity, supporting exposure to higher noise levels in rural/suburban areas (see Fig. 2). Furthermore, the influence of urbanicity increases with improving area-level socioeconomic conditions and increasing migration density, but in different directions. As area-level socioeconomic conditions improve, the noise levels become lower while as migration density increases, the noise levels slightly become higher. Regarding vibration, we observe a pattern of higher exposure in rural-suburban areas, but no interaction effects, evidenced by the parallel velocity predictions in Fig. 2.

## Discussion

Our findings indicate that railway noise and vibration are unequally distributed according to area-level socioeconomic conditions in Southwest Sweden. Areas with better socioeconomic conditions are consistently less exposed to both railway noise and vibration. Higher migration density was associated with increased vibration exposure. Only after accounting for spatial autocorrelation did the analysis reveal higher noise levels in areas with more than 20% of inhabitants with a foreign background. The impact of area-level indicators on noise exposure also depends on urbanicity, with higher exposure levels in rural and suburban areas compared to urban areas. This difference is more pronounced with improving socioeconomic conditions and decreasing migration density. Analyses suggest relevant spatial autocorrelation effects in both exposures and predictors, and mapping the exposures further illustrated the complexities in the distribution of noise, and especially of vibration, depending partially on the area-level social vulnerability. The results should not be interpreted as indicating that individuals with certain socioeconomic characteristics are necessarily more exposed, but rather that such exposures are patterned across areas with differing socioeconomic characteristics.

Previous studies suggest that railway noise is closely linked to area-level socioeconomic characteristics (Casey et al. 2017; Peris and Arguelles 2023; Tonne et al. 2018). Using a small-area analysis, Peris and Arguelles (2023) reported non-homogeneous patterns of socioeconomic inequalities in transport-related noise exposure across four urban areas in England. The strongest and most consistent evidence of higher railway noise exposure among more socioeconomically disadvantaged neighbourhoods was observed in Luton, whereas Crawley showed inverse patterns, with less deprived areas more exposed. In Manchester and Newcastle, associations were generally weak and mixed, depending on the socio-economic indicator considered and the noise threshold analysed. However, studies in cities such as Paris, Oslo, Manchester, and Newcastle—often relying on aggregated exposure indicators and correlation-based or spatial analyses—show that wealthier neighbourhoods can also experience high levels of railway noise (Fyhri and Klaeboe 2006; Havard et al. 2009; Peris and Arguelles 2023). These inconsistencies between studies and settings likely stem from methodological and contextual differences. Some of these differences are discussed in the paragraphs below.

For instance, crude indicators of social vulnerability like area-level median income may not fully capture the true extent of vulnerability, limiting our understanding of the drivers of inequities in railway exposures. Social vulnerability is complex, context-specific, and influenced by multiple axes of disadvantage and oppression (Evans 2019; Smith 2009). Our analysis also used relatively simple area-level indicators to explore processes of social vulnerability regarding the exposure distribution. Yet, we explored and conceptualised different dimensions of social vulnerability–area-level deprivation and migration-related residential segregation–showing that both are relevant for the differential railway noise and vibration exposures (Davoudi and Brooks 2014; Walker 2012).

Conceptually, area-level deprivation and segregation refer to different dimensions of social vulnerability (Smith 2009), although these dimensions overlap in several contexts. Deprivation typically involves a lack of resources and opportunities, encompassing factors such as income and employment. Segregation, on the other hand, involves the spatial separation of different demographic groups, often along racial or ethnic lines (Wacquant et al. 2014). The uneven distribution of noise exposure across areas with different migration density may be related to income inequalities as well as due to residential choice differences, for instance, valuing other aspects (e.g., the desire to live near the transport network) more than living in a quiet area (Casey et al. 2017; Namba et al. 1991).

Besides the findings that support the links between socioeconomic deprivation and noise distribution, evidence from Canada and the US points to higher noise exposure in more racially segregated communities (Carrier et al. 2016; Casey et al. 2017; Nega et al. 2013). The more pronounced residential segregation in North America compared to Europe may explain the observed positive association between higher noise levels and greater segregation in the former, but not in the latter. In Sweden, residential sorting by income in large cities is strongly associated with migration background. In addition, beyond economic power, in Sweden people often move in together with others from similar countries of origin, living in areas with a comparable population profile (Jarvis et al. 2023). Overall, it is conceivable that in European studies, researchers need conceptual clarity and contextually adequate indicators (Davoudi and Brooks 2014; Walker 2012) when characterising and exploring the effects of residential segregation on environmental health. In the literature, terms such as racial segregation, ethnic segregation, and migration-related segregation are often used interchangeably, despite referring to different phenomena (Smith 2009; Wacquant et al. 2014). This conflation creates conceptual ambiguity and risks obscuring the fact that multiple mechanisms—such as structural racism, cultural preferences and social cohesion, or discrimination in housing markets—can operate simultaneously (Casey et al. 2017). These challenges highlight the importance of striving not only for conceptual clarity but also for transparency in the mechanisms assumed when exploring the relationships between segregation and environmental health (Davoudi and Brooks 2014; Walker 2012).

Railway noise exposure is closely linked to social vulnerability due to its disproportionate presence in lower-income and marginalised communities. These areas tend to have lower property values, making them more accessible to populations in vulnerable situations (Day et al. 2007). In addition, the residents in these areas often lack awareness as well as the political and financial resources to make complaints, to oppose infrastructure projects or to implement mitigation measures (Yılmazer et al. 2025). Evidence from Canada suggests that noise mitigation infrastructure itself may be unequally distributed. For example, Potvin et al. (2019) showed that noise barriers and acoustic mounds are less likely to be present in areas experiencing higher noise levels, potentially reinforcing environmental inequities. Assessing whether similar inequities exist in the allocation of railway noise mitigation measures in Sweden warrants further investigation.

Acoustics may also play a role. Unlike road traffic noise, railway noise includes low-frequency components and vibrations that penetrate buildings more effectively and are harder to mitigate (International Union of Railways (UIC) 2017; International Union of Railways (UIC) 2022; WHO 2018). The perceived uncontrollability and unpredictability of train noise (especially at night) can also play a role, potentially affecting residential choice processes (Namba et al. 1991; Seidler et al. 2023). Regulatory frameworks for railway noise are generally weaker, and mitigation strategies are inconsistently applied, particularly in older or underfunded rail networks (Yılmazer et al. 2025). These factors contribute to heightened perceptions of injustice and stress among affected populations, potentially reinforcing existing health and social inequities.

On the contextual differences—at country, city and urbanicity levels—there are multiple and diverse intersections between socioeconomic factors and environmental exposures that could explain differential exposure according to varying social vulnerability. These intersections may be related to transport planning, housing policies, urban design, residential choice processes, and collective efficacy in how communities mobilize to address environmental hazards (Peris and Arguelles 2023; Tonne et al. 2018). Contrary to other studies (Vienneau et al. 2019), we report higher noise and vibration exposure in rural and suburban areas compared to urban areas. Living in areas with good and very good socioeconomic conditions and with lower migration density in rural areas does not give the same advantages as in urban areas regarding railway noise. This could be related to the structural differences between urban and rural areas regarding access to services and to authorities’ attention as well as to residential choice processes including identity feelings (e.g., emplacement) (Davoudi and Brooks 2014; Peris and Arguelles 2023; Wacquant et al. 2014; Walker 2012).

Previous studies have discussed the importance of taking spatial autocorrelation into account when analysing environmental inequalities (Carrier et al. 2016; Elford and Adams 2021; Havard et al. 2009; Iungman et al. 2021; Verbeek 2019). In Ghent, a study on air pollution and road traffic noise reports that spatial regression models performed better when compared to OLS linear regression models. In addition, income levels were only significant for explaining noise exposure using spatial regression models, but not linear regression models (Verbeek 2019). We report similarly in relation to migration density and noise distribution. These findings suggest that models that account for spatial factors capture better the overall impact of migration density across the entire railway line and not only limited to localized overlaps between higher migration density and higher railway noise levels.

Regarding railway vibration, this study is the first globally to examine inequalities in its distribution. We found that areas with better socioeconomic conditions and lower migration density were less exposed to vibration. We propose that these inequalities in vibration exposure may explain in part the inconsistencies in the literature regarding inequalities in railway noise exposure. Although noise and vibration are correlated, their propagation differs, and it is possible to have areas exposed to both or only to noise (International Union of Railways (UIC) 2022). Since vibration is less common and mostly perceived in houses rather than apartment buildings, the pattern of inequalities might differ from those observed for railway noise (Peris and Arguelles 2023; Tonne et al. 2018). Vibration propagates through the ground and building structures, possibly making it more noticeable in houses with direct ground contact compared to apartment buildings, which might have intrinsic structural isolation or alternatively might have structure resonances at higher floors (International Union of Railways (UIC) 2017; Ögren et al. 2021). This difference in propagation can lead to varied exposure patterns, with houses in deprived areas experiencing more vibration due to poorer construction standards and closer proximity to rail lines (International Union of Railways (UIC) 2017), while people living in apartments even in more deprived or segregated areas might not be as exposed to vibrations. Yet further studies are necessary regarding these patterns of vulnerability, housing types and vibration exposure.

These inequalities in railway noise and vibration distribution could have consequences for health equity, particularly when higher exposure coincides with population groups that may also be more susceptible to the effects of noise (Vincens et al. 2026). Indeed, these findings support environmental justice concerns, particularly regarding unequal exposure of railway hazards. However, exposure alone does not capture the full scope of injustice (Davoudi and Brooks 2014; European Environment Agency 2018; Walker 2012). Future research should explore how differential susceptibility and capacity to respond—shaped by health status, socioeconomic resources, residential choice profiles, social practices and material and immaterial structures—affect health equity (Vincens et al. 2026). Vulnerable populations may face compounded risks due to differential exposure, susceptibility factors (e.g., age, pre-existing health conditions, status syndrome) and limited means to cope with noise exposure and its impacts on health, such as access to well-insulated housing or use of restoration areas and practices. In contrast, more affluent groups often have greater capacity to shield themselves from environmental stressors (Evans 2019; Walker 2012). Mapping spatial and social disparities is only a starting point; a comprehensive environmental justice approach requires integrating data analysis with individual- and community-driven perspectives (Davoudi and Brooks 2014; Walker 2012).

### Implications and future research

From a practical perspective, these findings highlight the importance of incorporating area-level socioeconomic conditions, migration density, and urbanicity into railway noise and vibration assessment and mitigation strategies. Identifying areas where higher exposure coincides with social vulnerability may help prioritise interventions, support a more equitable allocation of mitigation measures, and inform transport planning and environmental health policy (Vincens et al. 2026). Such considerations are particularly relevant in the context of expanding rail infrastructure and sustainability-driven transport policies, where the benefits of rail transport need to be balanced against potential inequities in environmental exposure.

The EpiVib project also includes individual-level survey data on perceptions and annoyance related to railway noise and vibration (Maclachlan et al. 2018), which were not analysed in the present study. These data offer opportunities for research to examine how objectively modelled exposure relates to subjective experiences of noise and vibration (Maclachlan et al. 2018) as well as health outcomes, and whether such relationships vary across socioeconomic contexts or urban–rural settings (Vincens et al. 2026). Integrating spatial exposure patterns with perception-based and health-related outcomes in future analyses would allow a more comprehensive assessment of environmental equity, encompassing not only differential exposure but also differential experience and response.

### Limitations

The EpiVib project had a low response rate, which we attributed to declining trends in participation observed across all fields of health research in Sweden. Low response rates are a well-documented challenge in socio-acoustic and transportation noise research. Recent work by Pinsonnault-Skvarenina et al. (2025) shows that participation in noise-related surveys is influenced by several factors, including survey fatigue, perceived relevance of the exposure, trust in authorities, and attitudes toward transportation infrastructure. These factors may partly explain the response rate observed in the present study and are consistent with broader trends of declining participation in environmental health surveys.

An analysis of non-respondents based on demographic data revealed that respondents were older (mean age: 56.2 vs. 48.2 years, *p* < 0.001) and lived closer to the railway (mean distance: 409 m vs. 460 m, *p* < 0.001). These differences suggest a potential overrepresentation of individuals with higher exposure levels, which may lead to a slight overestimation of absolute exposure levels and possibly of exposure inequalities. Additionally, the low response rate in EpiVib could be partially due to the positive perception and attitude towards rail transportation among the individuals invited for the study as suggested in previous socio-acoustic studies (Pinsonnault-Skvarenina et al. 2025). This might result in an overrepresentation of people who are displeased and potentially disturbed by rail traffic. Conversely, individuals less affected by noise may have been less likely to participate, which could further reinforce this bias. However, previous analyses comparing our estimates with those of other studies indicate that our sample should not be excessively negative towards rail (Maclachlan et al. 2018). Overall, while some degree of selection bias cannot be excluded, its impact on the observed patterns is likely limited.

While the EpiVib project includes individual-level data on noise and vibration perception as well as data on health outcomes, the present study focuses specifically on the spatial distribution of railway noise and vibration exposure and its association with area-level socioeconomic conditions and migration density. Analyses linking exposure to perceptions and health outcomes are addressed in separate publications within the broader research project (MacLachlan). Assessing differential susceptibility was beyond the scope of this analysis.

A significant limitation of our study is the considerable uncertainty in the predicted vibration levels. This uncertainty arises from the complex nature of ground vibration generation and propagation, as well as variability in input data. For instance, geological maps provide information on soil type and thickness, indicating predominant soil types like clay at certain depths. However, local variations can occur, and test drilling at specific sites might yield different results (Ögren et al. 2021). The method we used for noise calculation is the standard and official noise prediction method in use in the Nordic countries and has been validated using measurements across all the Nordic countries. The method is also updated when new train types enter service. Such modelling approaches are widely applied and recommended for population-based assessments of transportation noise exposure (European Environment Agency 2020; WHO 2018).

## Conclusion

We report inequalities in railway noise and vibration exposure across different socioeconomic and demographic areas in Southwest Sweden. There are still few studies on railway noise inequalities and to our knowledge none on vibration. Areas with better socioeconomic conditions are consistently less exposed to both railway noise and vibration. Higher migration density was associated with increased vibration exposure although only after accounting for spatial autocorrelation did the analysis reveal higher noise levels in these areas. The impact of area-level indicators on noise exposure also depends on urbanicity degree, with higher exposure levels in rural and suburban areas compared to urban areas. Interventions and policies that account for area-level socioeconomic factors as well as urban and rural contexts are advised to ensure environmental equity and justice regarding rail traffic exposures.

## Supplementary Information

Below is the link to the electronic supplementary material.

ESM 1 (DOCX 19.4 KB)

## Data Availability

The datasets generated and/or analysed during the current study are not publicly available due to the sensitive nature of the data but are available from the corresponding author on reasonable request and with appropriate ethical approval.

## Declarations

### Clinical trial registration

Clinical trial number: not applicable.
