# Supplementary material for: Exposure differences in railway noise, vibration, area-level socioeconomic conditions and migration density: spatial autocorrelation and urbanicity impacts in Southwest Sweden
Source: Environ Sci Pollut Res Int. 2026 Jul 2;33(21):10499–512. doi: 10.1007/s11356-026-37951-1 (PMC13369225; doi:10.1007/s11356-026-37951-1)
Supplement: Supplementary file 1 — (DOCX 19.4 KB) [file 11356_2026_37951_MOESM1_ESM.docx]

## Supplementary material

Supplementary Table S1. Number and percentage of study participants by distance to the railway in 100 m intervals.

| **Distance to the rail in meters** | **n** | **%** |
| --- | --- | --- |
| 0-100 | 721 | 9.9 |
| 100-200 | 1111 | 15.26 |
| 200-300 | 1184 | 16.26 |
| 300-400 | 1061 | 14.57 |
| 400-500 | 787 | 10.81 |
| 500-600 | 575 | 7.9 |
| 600-700 | 517 | 7.1 |
| 700-800 | 502 | 6.9 |
| 800-900 | 432 | 5.93 |
| 900-1000 | 390 | 5.36 |
| Total | 7280 | 100 |
